# Advancements in Glioblastoma Multiforme Treatment: A Comprehensive Systematic Review and Meta‐Analysis

**DOI:** 10.1002/brb3.71456

**Published:** 2026-04-28

**Authors:** Kayode Agboola, Fatemeh Khafaji, Bipin Chaurasia, Kashif Qureshi, Kivanc Yangi, Vishal Chavda, Naci Balak

**Affiliations:** ^1^ Facharztzentrum for Surgery and Orthopaedics Essen Germany; ^2^ Department of Neurosurgery, Romodanov Institute of Neurosurgery National Academy of Medical Sciences of Ukraine Kyiv Ukraine; ^3^ Department of Neurosurgery Medical Campus Oberfranken of FAU Erlangen Bayreuth Germany; ^4^ Department of Neurosurgery College of Medical Sciences Bharatpur Nepal; ^5^ Department of Neurosurgery Yale School of Medicine New Haven Connecticut USA; ^6^ Department of Neurosurgery Barrow Neurological Institute Phoenix Arizona USA; ^7^ Department of Medicine and Critical Care, Multispecialty, Trauma and ICCU Center Sardar Hospital Ahmedabad Gujarat India; ^8^ Department of Neurology, Neurosurgery and Neurogenetics, Department of Clinical and Translational Research ChromoMed Institute Santo Domingo Dominican Republic; ^9^ Department of Neurosurgery Istanbul Medeniyet University Istanbul Türkiye

**Keywords:** adverse events, bevacizumab (BEV), glioblastoma multiforme (GBM), lomustine (CCNU), meta‐analysis, overall survival (OS), progression‐free survival (PFS), temozolomide (TMZ)

## Abstract

**Background:**

Glioblastoma multiforme (GBM) poses significant challenges in oncology, with limited treatment options and poor prognosis. Despite advancements, recurrence remains common, emphasizing the need for effective therapeutic strategies. This meta‐analysis comprehensively evaluates the efficacy and safety of treatments like bevacizumab (BEV), temozolomide (TMZ), and lomustine (CCNU) for recurrent GBM (rGBM), aiming to identify optimal approaches and guide clinical decisions.

**Methods:**

Following Preferred Reporting Standards for Systematic Reviews and Meta‐Analyses (PRISMA) guidelines, a systematic review was conducted, and randomized controlled trials assessing interventions for rGBM were included. The Cochrane risk‐of‐bias assessment was used to evaluate study quality. Primary outcomes included progression‐free survival (PFS) and overall survival (OS), whereas adverse events were assessed as secondary outcomes.

**Results:**

Nine articles met the inclusion criteria, comprising a total of 1689 participants. BEV therapy outperformed pre‐BEV treatment across several parameters, including full resection (odds ratio [OR] = 14.50, 95% confidence intervals [CI]: 1.82–115.29, *p* = 0.01) and biopsy outcomes (OR = 18.67, 95% CI: 2.55–136.41, *p* = 0.04). BEV also demonstrated higher rates of MGMT promoter methylation at baseline (OR = 11.84, 95% CI: 1.87–74.77, *p* = 0.009), as well as associations with improved PFS (OR = 1.16, 95% CI: 0.10–2.22, *p* = 0.03) and OS (OR = 0.63, 95% CI: 0.01–1.26, *p* = 0.05) compared to pre‐BEV therapies. BEV monotherapy was associated with more favorable outcomes than combination therapies, with a pooled OR of 19.50 (95% CI: 2.69–141.35, *p* = 0.03) for corticosteroid use. Adverse event analysis revealed a lower incidence of CNS hemorrhage with standard therapy. TMZ outperformed the TMZ + CCNU combination in PFS (OR = 2.44, 95% CI: 1.23–4.83, *p* = 0.01), OS, and MGMT methylation (OR = 2.58, 95% CI: 1.40–4.78, *p* = 0.002).

**Conclusion:**

BEV monotherapy emerges as a promising treatment for rGBM, with favorable outcomes in biopsy results, corticosteroid use, PFS, and OS. Future research is needed to confirm these findings and optimize BEV's clinical application, emphasizing tailored treatment strategies to improve patient prognosis.

## Graphical abstract

This study is a systematic review and meta‐analysis comparing bevacizumab‐, temozolomide‐, and lomustine‐based treatments for recurrent glioblastoma. The findings suggest that monotherapy, particularly bevacizumab, may provide better clinical outcomes with fewer adverse events than combination regimens.

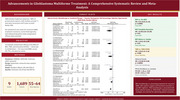

## 1 Introduction

Glioblastoma multiforme (GBM) presents a formidable challenge in oncology, constituting approximately 80% of primary brain tumors (Iyer et al. 2023). Designated as a Grade IV glioma according to the World Health Organization (WHO) classification, GBM is renowned for its aggressive characteristics and grim prognosis, especially in the adult population (Gupta and Dwivedi 2017). GBM strikes individuals across all age brackets, with a yearly occurrence rate of 3.2 instances per 100,000 persons (van Linde et al. 2017). Despite considerable progress in therapeutic approaches, almost all GBM patients experience relapse, with a median survival of only about 1‐year post‐recurrence (Cloughesy et al. 2019).

Detecting recurrence in GBM remains challenging, underscoring the intricate nature of managing this disease. Treatment resistance, tumor heterogeneity, and the dynamic evolution of subclonal populations within the tumor present significant hurdles in therapeutic interventions (Schritz et al. 2021). Presently, therapeutic treatments for GBM are scarce, lacking a clearly defined standard of care. Clinical trials play a pivotal role in guiding treatment decisions for recurrent GBM (rGBM) patients, considering factors such as performance status, tumor characteristics, and prior treatment history (Djamel‐Eddine et al. 2019).

Reoperation, although feasible for only a small fraction of patients, may offer survival advantages depending on variables such as performance status, tumor location, and age at relapse (Chaul‐Barbosa and Marques 2019). Another potential option is re‐irradiation, facilitated by advancements in imaging technology, albeit subject to stringent criteria (Chaul‐Barbosa and Marques 2019). Systemic treatments, primarily comprising alkylating agents like temozolomide (TMZ) and nitrosoureas such as carmustine (BCNU) or lomustine (CCNU), constitute another cornerstone in managing rGBM (Qi et al. 2016). Remarkably, TMZ is extensively utilized in managing both recently detected and recurring cases of GBM, whereas nitrosoureas, such as BCNU and CCNU, have shown effectiveness in this scenario (Gupta et al. 2023). Notably, bevacizumab (BEV), an anti‐angiogenic agent, holds FDA approval for rGBM treatment but lacks EMA endorsement due to insufficient data (Fu et al. 2016).

Despite several studies exploring various treatment options for rGBM, a thorough overview of all known medications in randomized controlled trials (RCTs) is strikingly lacking (Cote et al. 2017; Scherm et al. 2023). Thus, our study aims to bridge this gap by conducting an exhaustive systematic review to elucidate the relative efficacy of diverse treatment regimens. In particular, our aim is to offer an exhaustive examination of therapeutic alternatives evaluated for rGBM and gauge their relative effectiveness. Vital metrics like progression‐free survival (PFS) and overall survival (OS) will steer our evaluation. By amalgamating data from various trials, our research strives to provide invaluable perspectives on enhancing therapeutic approaches for this relentless illness.

## 2 Materials and Methods

Adhering to the guidelines outlined in the Preferred Reporting Standards for Systematic Reviews and Meta‐Analyses (PRISMA), the meta‐analysis concentrated on assessing the effectiveness of interventions for GBM (Scherm et al. 2023). Given its nature without involvement in human or animal experiments, ethical approval was deemed unnecessary.

### 2.1 PICO Inquiry

Within the cohort of adult individuals (aged ≥18) who were diagnosed with rGBM, the effectiveness of treatments such as alkylating agents like TMZ or nitrosoureas, such as BCNU, BEV, or CCNU, either alone or in combination with other modalities, in addressing rGBM, was scrutinized against either placebo or an active comparator in terms of OS, PFS, and tumor response rates.

### 2.2 Eligibility Criteria

Randomised controlled trials enrolling adults (>18 yrs) with histologically confirmed recurrennt glioblastoma were included if they evaluated bevacizumab, temozolamide or lomustine (alone or in combination) against active comparator therapies and reported survival or response outcomes.

### 2.3 Inclusion Criteria

- **Population**: RCTs involving adult patients (aged ≥18) with rGBM.
- **Intervention**: Studies evaluating treatments such as alkylating agents (e.g., TMZ), nitrosoureas (e.g., BCNU), BEV, or CCNU, either alone or in combination with other therapies.
- **Comparator**: Studies comparing the aforementioned therapies to either an inert control (placebo) or an active comparator (reference therapy).
- **Outcomes**: Studies reporting on OS, PFS, or tumor response rates.

### 2.4 Exclusion Criteria

- **Animal studies**: Studies conducted on animals were excluded to ensure that the review is focused on human clinical data.
- **Non‐GBM cancer patients**: Studies involving patients with other types of cancer or disorders were excluded to maintain the specificity of the findings to rGBM.
- **Reanalyzed RCTs**: Studies that reanalyzed previously conducted RCTs without presenting new primary data were excluded to avoid redundancy and potential bias.
- **Non‐randomized allocation**: Studies that did not utilize randomized allocation of treatments were excluded to ensure the robustness and reliability of the comparative data.
- **Publication type**: Studies published solely as abstracts, reviews, editorials, or letters were excluded due to insufficient detail and lack of peer‐reviewed data.
- **Nonhuman subjects**: Any study not involving human subjects was excluded to ensure the applicability of the findings to clinical practice.

### 2.5 Literature Search Approach

The search strategy encompassed a comprehensive exploration of literature published from 2017 onwards across EMBASE, MEDLINE, and the Cochrane Research Register. It utilized a range of keywords related to glioblastoma (e.g., glioblastoma, GBM, and glioma), chemotherapy agents (e.g., TMZ and BEV), and study designs (e.g., randomized and RCT). Boolean operators (AND, OR) were strategically applied to enhance search effectiveness, supplemented by other relevant articles and materials to ensure thoroughness in literature identification.

### 2.6 Study Selection

**FIGURE 1:**
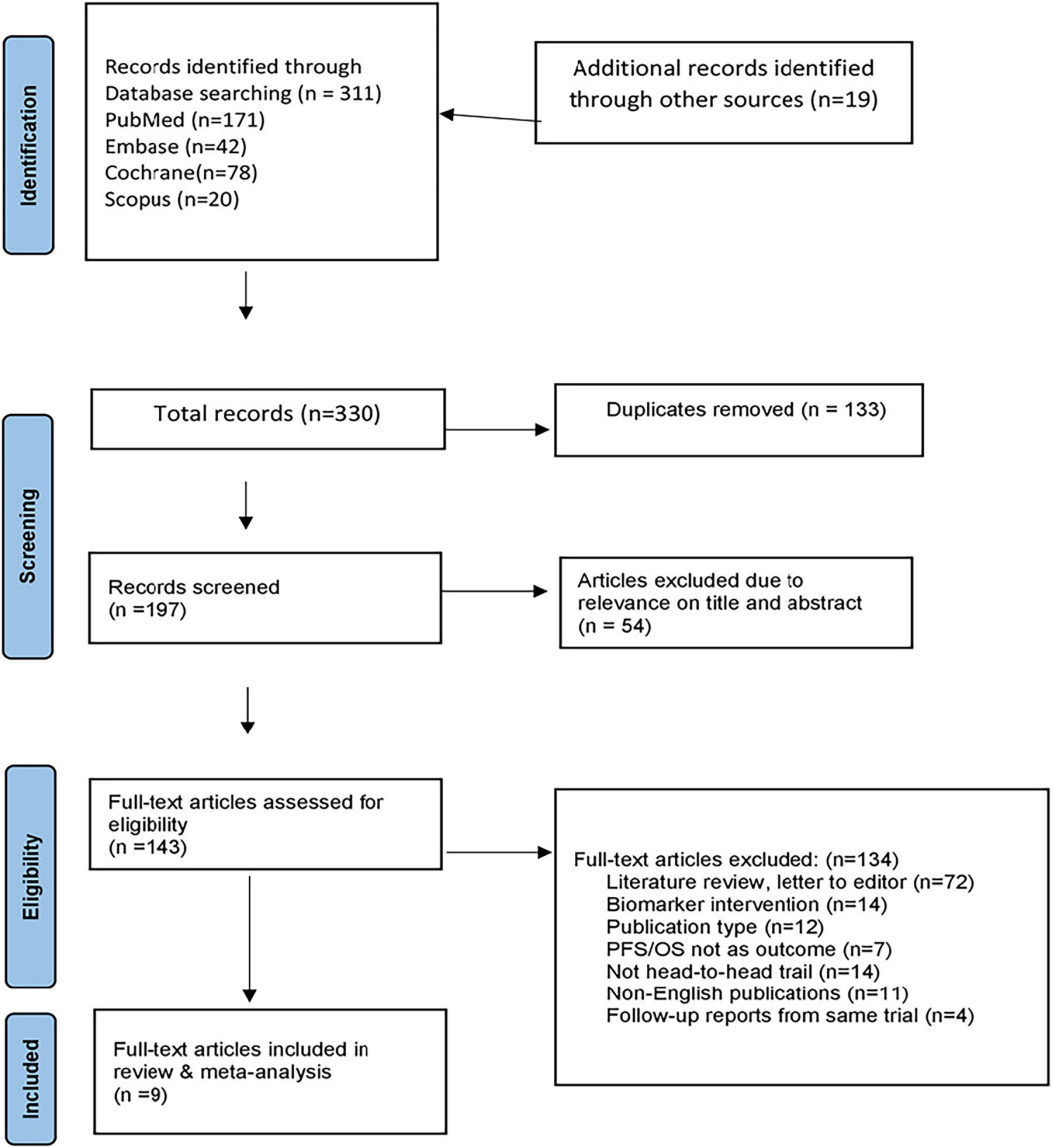
PRISMA flowchart of the studies. Software: Microsoft Corporation. Microsoft Word 2019. Redmond, Washington, USA. OS, overall survival; PFS, progression‐free survival. *Source*: M. J. Page et al. (2021).

Each article was rigorously screened by two independent reviewers based on title and abstract, resolving disagreements through discussion. Potentially eligible studies underwent full‐text screening, with conflicts resolved by a third reviewer if necessary. Additional trials were identified through hand‐searching systematic reviews (Figure 1).

### 2.7 Data Extraction

Relevant data, including study approach, participant characteristics, sample sizes, specifics of interventions and controls, duration of observation, measures of survival, and adverse events, were meticulously collected using a standardized framework organized according to the PICOS structure. Information retrieval was carried out independently by two assessors, with any disparities resolved through collaborative deliberation.

### 2.8 Primary Outcomes and Quality Assessment

**FIGURE 2:**
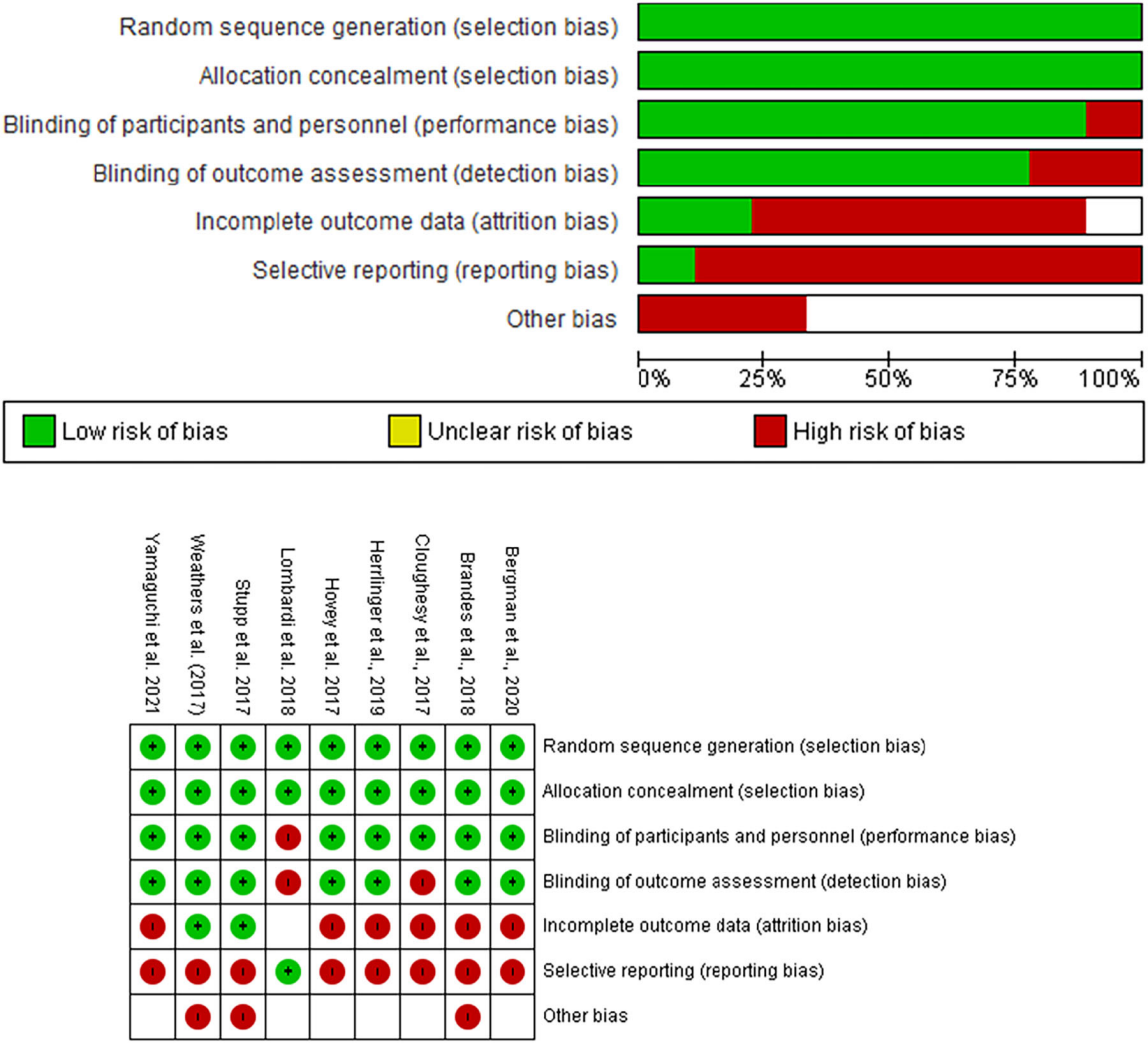
Risk of bias for nine studies included in the meta‐analysis.

The primary emphasis of the study was comparing the efficacy of PFS and OS, whereas ancillary aims included examining the adverse effects of adjuvant TMZ, BEV, and other therapies. OS was computed from the initiation of treatment (surgery) or study commencement until the final follow‐up or death from any cause, whereas PFS was assessed from treatment initiation or study commencement to documented clinical or radiographic progression, final follow‐up, or death from any cause, whichever occurred first. Assessment of toxicity involved evaluating Grade 3 or higher myelotoxicity induced by TMZ during adjuvant therapy. Table 1 illustrates quality assessment utilizing the Cochrane risk of bias tool for RCTs, examining seven domains: random sequence generation, allocation concealment, blinding of participants and personnel, blinding of outcome assessment, incomplete outcome data, selective reporting, and other biases (Figure 2).

**TABLE 1:** Cochrane risk of bias assessment for each included study.

Study	Random sequence generation	Allocation concealment	Blinding of participants and personnel	Blinding of outcome assessment	Incomplete outcome data	Selective reporting	Other bias
Yamaguchi et al. (2021)	Low risk	Low risk	Low risk	Low risk	High risk	High risk	High risk
Hovey et al. (2017)	Low risk	Low risk	Low risk	Low risk	High risk	High risk	High risk
Cloughesy et al. (2017)	Low risk	Low risk	Low risk	High risk	High risk	High risk	High risk
Brandes et al. (2018)	Low risk	Low risk	Low risk	Low risk	High risk	High risk	High risk
Lombardi et al. (2019)	Low risk	Low risk	High risk	High risk	Unclear risk	Low risk	Unclear risk
Weathers et al. (2020)	Low risk	Low risk	Low risk	Low risk	Low risk	High risk	High risk
Herrlinger et al. (2019)	Low risk	Low risk	Low risk	Low risk	High risk	High risk	Unclear risk
Stupp et al. (2017)	Low risk	Low risk	Low risk	Low risk	Low risk	High risk	High risk
Bergman et al. (2020)	Low risk	Low risk	Low risk	Low risk	High risk	High risk	Unclear risk

*Note*: Key to risk of bias levels. Low risk: Adequate measures were taken to minimize bias. Unclear risk: Insufficient information provided to assess the risk of bias. High risk: Evidence suggests that the presence of bias may have influenced the results.

### 2.9 Meta‐Analysis

To account for predicted variability, a random‐effects model was used to examine data using Review Manager version 5.3. Continuous data were given as standardized mean differences (SMD) with 95% confidence intervals (CI), whereas categorical outcomes were shown as odds ratios (ORs) with 95% CI. We acknowledge that for time‐to‐event outcomes such as PFS and OS, hazard ratios (HRs) derived from individual patient‐level data or reported Kaplan–Meier statistics represent the methodologically preferred summary measure. However, the included studies did not consistently report HRs or the log‐HR and standard error (SE) required for pooling; several studies reported only dichotomized survival proportions at fixed time points or median survival values without accompanying variance estimates. Under these data constraints, ORs were used as an approximation for the relative comparison of binary survival thresholds across studies, following a precedent established in meta‐analyses of rGBM literature.

### 2.10 Assessment of Statistical Heterogeneity

Statistical heterogeneity among the included studies was assessed using Cochran's *Q* test and the *I* ^2^ index. The significance level for Cochran's *Q* test was set at *p* < 0.10, which is less stringent than typical significance levels to detect heterogeneity more sensitively. The *I* ^2^ index, which describes the percentage of total variation across studies due to heterogeneity rather than chance, was also reported. An *I* ^2^ value greater than 50% was considered indicative of substantial heterogeneity. When substantial heterogeneity was detected (*I* ^2^ > 50%), potential sources were explored through subgroup analyses and sensitivity analyses. Subgroup analyses were conducted on the basis of variables, such as the type of intervention, patient demographics, and study quality. Sensitivity analyses involved excluding studies with high risk of bias or those that significantly deviated from others in terms of results or methodology.

### 2.11 Publication Bias

To assess publication bias, both forest plots and funnel plots were employed. Forest plots provided a visual summary of the individual study estimates and their precision. Funnel plots were used to detect asymmetry, which can suggest the presence of publication bias. Funnel plots were created by plotting the effect size of each study against its SE. In the absence of publication bias, the studies’ results are expected to scatter symmetrically about the combined effect size, forming an inverted funnel shape. To quantify the degree of asymmetry, Egger's regression test was conducted. This statistical test examines the relationship between the SE and the effect size, with a *p* value <0.05 indicating significant asymmetry and, consequently, potential publication bias.

By detailing the approach to assessing and managing heterogeneity, this meta‐analysis aims to provide a thorough and transparent evaluation of the data, enhancing the robustness of the conclusions drawn about the effectiveness of treatments for rGBM.

## 3 Results

### 3.1 Characteristics of the Studies

Out of 330 records identified, 197 were screened, and 143 full‐text articles were assessed for eligibility. Among these, 134 were excluded for various reasons, including literature reviews, letters to the editor, and publications that did not focus on head‐to‐head trials or were non‐English. Finally, nine articles were included in the review and meta‐analysis, covering a range of topics related to the desired outcomes, such as PFS and OS, as shown in Figure 1. It should be noted that although the primary focus of this review is rGBM, two included trials, Stupp et al. (2017) and Herrlinger et al. (2019), enrolled newly diagnosed GBM patients. These trials were retained because they evaluated treatment regimens (TTFields plus TMZ; CCNU–TMZ combination) that are directly relevant to understanding the comparative efficacy of agents used across the GBM treatment continuum and inform management decisions at recurrence. Analyses involving these studies are clearly distinguished, and separate subgroup considerations for newly diagnosed versus rGBM are addressed where applicable in the results and discussion.

### 3.2 Study Participants and Baseline Characteristics

**FIGURE 3:**
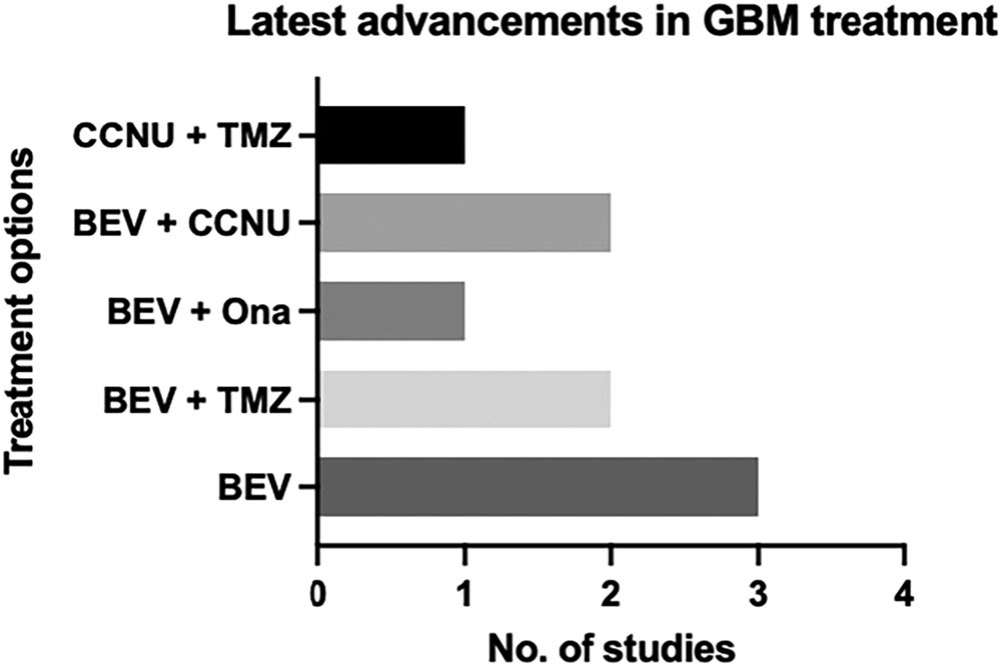
A graphical representation of latest advancements used in glioblastoma multiforme (GBM) treatment in the selected studies titled Latest advancements in GBM treatment. BEV, bevacizumab; CCNU, lomustine; TMZ, temozolomide. *Source*: Schritz et al. (2021) and Scherm et al. (2023).

The study included 1689 participants across multiple research settings, with a mean age ranging from 55.2 to 64 years. Gender distribution varied across studies, with the majority being male (78%). Table 2 highlights that most studies on glioblastoma treatments are designed as Phase II trials, with a primary focus on tracking patient outcomes until disease progression or death. Follow‐up rates are generally high, with Phase II studies prioritizing long‐term monitoring to evaluate treatment efficacy. In Phase III studies, follow‐up extends even further, with durations of up to 36 months or a median of 40 months, reflecting the need for extended observation. Additionally, some studies, such as Bergman et al. (2020), include regular imaging (e.g., every 2 months) to closely monitor disease progression. Figure 3 represents the latest advancements in the treatment of GBM. The extent of resection varied, with a significant proportion undergoing gross total resection (82%). Treatment strategies included various combinations of chemotherapy, radiotherapy, and targeted therapies, administered at specified doses. The mean PFS ranged from 1.8 to 16.7 months, and the mean OS ranged from 2.9 to 20.4 months. The overall follow‐up period ranged from 36 months until disease progression or death, depending on the study design, as shown in Table 3.

**TABLE 2:** Clinical phase and follow‐up duration in glioblastoma multiforme studies.

Study	Clinical phase	Follow‐up
Yamaguchi et al. (2021)	Retrospective II	Until progression or death
Hovey et al. (2017)	Phase II	Until progression or death
Cloughesy et al. (2017)	Phase II	Until progression
Brandes et al. (2018)	Phase II	Until second PD
Lombardi et al. (2019)	Phase II	Median: 15.4 months
Weathers et al. (2020)	Phase II	Until progression or death
Herrlinger et al. (2019)	Phase III	Up to 36 months
Stupp et al. (2017)	Phase III	Median: 40 months
Bergman et al. (2020)	Phase II	Brain MRI every 2 months

**TABLE 3:** Data extraction sheet of the selected nine studies.

Author name and year of publication	Study location	Population characteristics	Clinical phase	Treatment strategy	Dose of treatment	Follow‐up	Mean PFS value	Mean OS value	Main findings
Yamaguchi et al. (2021)	Hokkaido University Hospital and Kashiwaba Neurosurgical Hospital, Sapporo, Hokkaido, Japan	Total of 157 primary patients with GBM (90 males, 67 females); median age of onset: 64 years (range, 25–85 years); extent of resection at primary surgery: 11.5% biopsy, 17.2% partial resection, 27.4% subtotal resection, 43.9% gross total resection	Retrospective analysis phase II	Conventional radiotherapy concomitant with TMZ according to the Stupp protocol for GBM; BEV added for continuously progressing tumors during radiotherapy from June 2013; adjuvant TMZ chemotherapy (150–200 mg/m^2^, 5 days, every 4 weeks) for 12–24 courses; contrast‐enhanced MRI every 3 months during and after adjuvant TMZ chemotherapy	Radiotherapy: 60 Gy/30 Fr localized, TMZ chemotherapy: 150–200 mg/m^2^, 5 days, every 4 weeks	Median OS: 20.4 months; 2‐year OS: 39.3%	Pre‐BEV group: 4.2 months; BEV group: 4.9 months	Pre‐BEV group: 8.1 months; BEV group: 16.3 months	Cytoreductive surgery prior to BEV significantly prolonged overall survival after first recurrence in patients with recurrent GBM, especially in the BEV era. Postoperative availability of BEV and maintenance of Karnofsky performance status after surgery were significant prognostic factors for survival
Hovey et al. (2017)	Cooperative Trials Group for Neuro‐Oncology (COGNO), coordinated at the National Health and Medical Research Council (NHMRC) Clinical Trials Centre, University of Sydney, Australia	Adults (age range: 25–82 years) with recurrent glioblastoma after receiving radiotherapy and temozolomide, ECOG performance status 0–2, with various resection phases, including biopsy, debulking, and resection	Phase II	Randomized patients to continue or cease bevacizumab beyond progression; additional chemotherapy regimens (carboplatin, temozolomide, etoposide) or best supportive care	Bevacizumab 10 mg/kg intravenously 2‐weekly; carboplatin AUC 5 every 4 weeks; etoposide 50 mg/m^2^ daily for 20 days every 28 days; temozolomide 150–200 mg/m^2^ daily for 5 days every 28 days	Until disease progression or death	Bevacizumab continuation: 1.8 months; Bevacizumab cessation: 2.0 months	Bevacizumab continuation: 3.4 months; bevacizumab cessation: 3.0 months	No significant difference in PFS or OS between continuing or ceasing bevacizumab beyond progression; no radiological responses observed; no clear survival benefit with continuation; bevacizumab continuation not associated with better quality of life
Cloughesy et al. (2017)	Multicenter study conducted across 42 centers in eight countries	Recurrent glioblastoma patients (total *n* = 129); age range: 55 years; gender distribution: 44 males, 20 females; resection status: biopsy, debulking	Phase II	Random assignment to receive either onartuzumab (15 mg/kg) plus bevacizumab (15 mg/kg) or placebo plus bevacizumab, administered in 3‐weekly cycles	Onartuzumab: 15 mg/kg; bevacizumab: 15 mg/kg	Until disease progression. 8.9–9.9 months	Onartuzumab plus bevacizumab: 3.9 months; placebo plus bevacizumab: 2.9 months	Onartuzumab plus bevacizumab: 8.8 months; placebo plus bevacizumab: 12.6 months	No significant difference in progression‐free survival (PFS) or overall survival (OS) between onartuzumab plus bevacizumab and placebo plus bevacizumab groups; Grade ≥3 adverse events were reported in 38.5% of patients receiving onartuzumab plus bevacizumab and 35.9% of patients receiving placebo plus bevacizumab
Brandes et al. (2018)	Department of Medical Oncology, Italy	296 patients with recurrent glioblastoma—age ≥18 years—newly diagnosed with glioblastoma—histologically confirmed with surgical resection or biopsy	Phase II	Combination therapy with CCNU + BEV or CCNU + placebo through multiple lines of treatment	CCNU dose: 90 mg/m^2^ every 6 weeks (max 160 mg), increasing to 110 mg/m^2^ (max 200 mg) if no hematologic toxicity grade >1	Until second PD (progression of disease)	PFS2: CCNU + BEV: 2.3 months CCNU + placebo: 1.8 months PFS3: CCNU + BEV: 2.0 months CCNU + placebo: 2.2 months	CCNU + BEV: 6.4 months CCNU + placebo: 5.5 months	No survival benefit observed with continuing BEV through multiple lines of treatment for recurrent glioblastoma. No detriment observed either. High dropout rate during first‐line treatment led to study termination
Lombardi et al. (2019)	Italy	Total: 119 patients; age: regorafenib group: 54.8 years; lomustine group: 58.9 years; gender: regorafenib group: male (69%), female (31%); lomustine group: male (72%), female (28%); resection: 22% in regorafenib group, 23% in lomustine group	Phase II	Regorafenib 160 mg once daily for 3 weeks of each 4‐week cycle vs. lomustine 110 mg/m^2^ once every 6 weeks	Regorafenib: 160 mg once daily; lomustine: 110 mg/m^2^ once every 6 weeks	Median: 15.4 months	Regorafenib: 2.0 months (95% CI: 1.9–3.6) Lomustine: 1.9 months (95% CI: 1.8–2.1)	Regorafenib: 7.4 months (95% CI: 5.8–12.0) lomustine: 5.6 months (95% CI: 4.7–7.3)	Regorafenib showed significantly improved overall survival compared to lomustine (HR 0.50, 95% CI: 0.33–0.75, *p* = 0.0009). Progression‐free survival was longer in the regorafenib group (HR 0.65, 95% CI: 0.45–0.95, *p* = 0.022). Grade 3–4 adverse events occurred in 56% of patients in the regorafenib group and 40% in the lomustine group
S.‐P. Weathers et al. (2020)	Single center, Department of Neuro‐Oncology, University of Texas	Total patients: 71; age: ≥18 years; gender: male 44, females 27; previous recurrence: first—25, second—10	Phase II	Standard dose bevacizumab vs. low dose bevacizumab plus lomustine	Bevacizumab: 10 mg/kg every 2 weeks; low dose bevacizumab: 5 mg/kg every 3 weeks plus lomustine (90 mg/m^2^) every 6 weeks	Until disease progression or death	4.34 (low dose bevacizumab + lomustine; 4.11 (bevacizumab alone)	9.6 (low dose bevacizumab + lomustine; 8.3 (bevacizumab alone)	Low dose bevacizumab plus lomustine did not significantly improve PFS compared to standard dose bevacizumab. A trend toward longer median PFS was seen in patients with first recurrence on the combination therapy
Herrlinger et al. (2019)	17 German university hospitals	657 newly diagnosed glioblastoma patients with methylated MGMT promoter, age 18–70, KPS ≥70%	Phase III	Lomustine–temozolomide combination therapy vs. standard temozolomide therapy	Lomustine (100 mg/m^2^ on Day 1) plus temozolomide (100–200 mg/m^2^ per day on Days 2–6 of the 6‐week course) in addition to radiotherapy (59–60 Gy) vs. standard temozolomide chemoradiotherapy (75 mg/m^2^ per day concomitant to radiotherapy followed by six courses of temozolomide 150–200 mg/m^2^ per day on the first 5 days of the 4‐week course)	Up to 36 months	Median PFS was 16.7 months with temozolomide and 16.7 months with lomustine–temozolomide	Median OS was 5.7 months with temozolomide and 6.7 months with lomustine–temozolomide	Lomustine–temozolomide chemotherapy showed improved overall survival compared to standard temozolomide therapy in patients with newly diagnosed glioblastoma with methylated MGMT promoter. Caution is warranted due to the small size of the trial
Stupp et al. (2017)	North America, Europe, Republic of Korea, and Israel	Total: 695 patients median age: 56 years gender: 68% male resection: biopsy: 13%, partial resection: 34%, gross total resection: 53%	Phase III	TTFields plus maintenance temozolomide vs. temozolomide alone	TTFields: low‐intensity, 200 kHz frequency alternating electric fields; temozolomide: 150–200 mg/m^2^ for 5 days per 28‐day cycle (6–12 cycles)	Median: 40 months	6.7 months in TTFields group vs. 4.0 months in temozolomide group	20.9 months in TTFields group vs. 16.0 months in temozolomide group	Addition of TTFields to temozolomide resulted in significant improvement in PFS and OS compared to temozolomide alone. Mild‐to‐moderate skin toxicity was observed in TTFields group. No significant increase in systemic adverse events
Bergman et al. (2020)	Single‐institution, Department of Neurosurgery, Henry Ford Health System, USA	Total: 35 patients, age: mean 55.2 years (range 27–81), gender: female (29%), male (71%), diagnosis: anaplastic astrocytoma (11%), anaplastic oligodendroglioma (6%), glioblastoma (83%), initial resection: biopsy (20%), GTR (29%), STR (52%), upfront RT dose: 54 Gy (3%), 59.4 Gy (6%), 60 Gy (91%)	Phase II	Fractionated stereotactic radiosurgery (FSRS) plus BEV‐based chemotherapy vs. BEV‐based chemotherapy alone	FSRS: 32 Gy to GTV, 24 Gy to CTV (delivered in 4 fractions within 2 weeks); chemotherapy: BEV combined with irinotecan, etoposide, temozolomide, or carboplatin	Brain MRI every 2 months until progression or death	FSRS plus BEV: 5.1 months (95% CI: 4.1–6.2), BEV‐based chemotherapy alone: 1.8 months (95% CI: 1.2–2.8)	Overall median survival: 6.6 months (95% CI: 5.7–7.5); FSRS plus BEV: 7.2 months (95% CI: 6.1–8.1); BEV‐based chemotherapy alone: 4.8 months (95% CI: 1.7–7.6)	FSRS combined with BEV‐based chemotherapy improves local control (LC) and progression‐free survival (PFS) compared to BEV‐based chemotherapy alone in patients with recurrent high‐grade glioma (HGG) progressing on bevacizumab (BEV). Overall survival (OS) was better in the FSRS group, although not statistically significant

Abbreviations: BEV, bevacizumab; CCNU, lomustine; CI, confidence intervals; GBM, glioblastoma multiforme; HR, hazard ratio; OS, overall survival; PFS, progression‐free survival; TMZ, temozolomide.

### 3.3 BEV Outperforms Pre‐BEV in GBM Treatment

**FIGURE 4:**
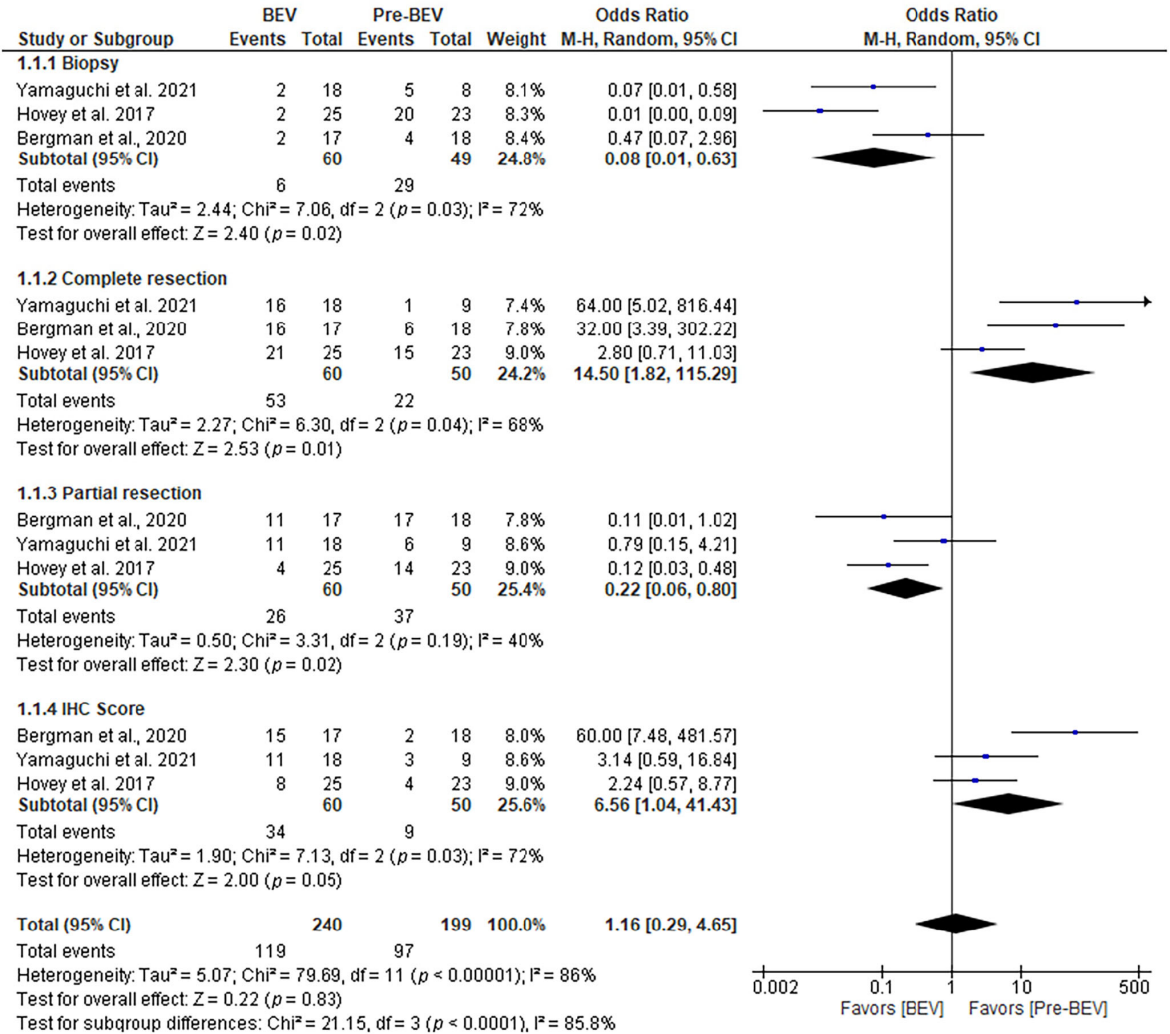
Forest plot showing the comparison between pre‐BEV and BEV therapy for the treatment of GBM patients, including biopsy‐related resection, complete resection, partial resection, and IHC score. Effect estimates are expressed as odds ratios (OR) with 95% confidence intervals (CI) using a random‐effects model. Statistical heterogeneity was quantified using the *I* ^2^ statistic and Cochran's *Q* test (significance threshold *p* < 0.10). BEV, bevacizumab.

The comparison between pre‐BEV and BEV therapies in patients with GBM revealed significant differences across several clinical parameters. In patients with biopsy‐only resection, the OR for favorable outcomes was 0.08 (95% CI: 0.01–0.63; *p* = 0.02), indicating significantly lower efficacy in the pre‐BEV group compared with BEV therapy. Complete resection outcomes favored BEV therapy, with an OR of 14.50 (95% CI: 1.82–115.29; *p* = 0.01), demonstrating its superior efficacy in enhancing complete tumor removal, likely due to its ability to reduce tumor vascularity and improve surgical outcomes. For partial resection, BEV therapy showed a significant advantage, with an OR of 0.22 (95% CI: 0.06–0.80; *p* = 0.02), suggesting a better response to residual tumor management compared with pre‐BEV therapy. Immunohistochemical (IHC) scoring also supported the superiority of BEV therapy, with an OR of 6.56 (95% CI: 1.04–41.43; *p* = 0.05), indicating improved expression of molecular markers associated with therapeutic response. Subgroup analyses demonstrated significant heterogeneity across most parameters (*I* ^2^ = 68%–72%), reflecting variability in patient outcomes, while consistently favoring BEV therapy. The overall effect size across all parameters was not statistically significant (OR = 1.16; 95% CI: 0.29–4.65; *p* = 0.83), likely due to high heterogeneity (*I* ^2^ = 86%). Nevertheless, BEV therapy consistently outperformed pre‐BEV treatment in critical outcomes, likely attributable to its targeted anti‐angiogenic effects, which enhance tumor control and improve surgical feasibility (Figure 4).

Analysis of baseline biomarker distribution between pre‐BEV and BEV‐treated groups showed that MGMT promoter methylation was more prevalent in the BEV group (OR = 11.84, 95% CI: 1.87–74.77, *p* = 0.009), as was IDH mutation status (OR = 10.39, 95% CI: 0.97–111.22, *p* = 0.05; *I* ^2^ = 75%). Importantly, MGMT promoter methylation and IDH mutation status are established pre‐treatment prognostic and predictive biomarkers determined at diagnosis; they are not endpoints influenced by therapy and should not be interpreted as treatment‐induced outcomes. These differences likely reflect baseline imbalances between patient cohorts rather than any biological effect of BEV on tumor molecular characteristics. Evaluation of KPS scores showed a pooled OR of 8.21 (95% CI: 1.32–50.98, *p* = 0.02), with BEV therapy associated with better performance status outcomes. Corticosteroid use was associated with an OR of 9.46 (95% CI: 1.32–67.94, *p* = 0.03), reflecting improved symptom management with BEV therapy. Overall, the total OR across all parameters was 9.21 (95% CI: 4.01–21.15, *p* < 0.00001), affirming the clinical benefits of BEV therapy over pre‐BEV therapy in GBM patients (Figure S1). The greater efficacy of BEV may be attributed to its targeted anti‐angiogenic effects, which reduce tumor vascularity and edema, thereby improving functional outcomes and reducing corticosteroid dependence. Publication bias was assessed using Egger's test, which indicated no significant bias (*p* = 0.15).

### 3.4 BEV Monotherapy Outperforms Combinations in GBM

**FIGURE 5:**
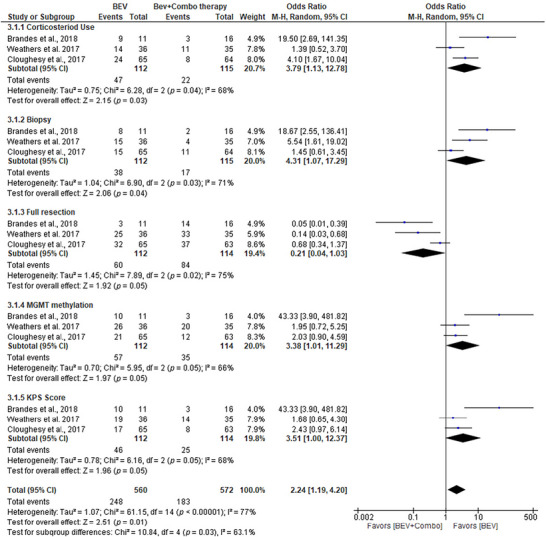
Forest plot of corticosteroid use, Karnofsky performance (KPS) value, and extent of resection among patients treated with mono bevacizumab (BEV) versus combined therapy. Effect estimates are expressed as odds ratios (OR) with 95% confidence intervals (CI) using a random‐effects model. Statistical heterogeneity was quantified using the *I* ^2^ statistic and Cochran's *Q* test.

In the comparison between BEV monotherapy and combination therapies (CCNU, TMZ, and onartuzumab) in the treatment of GBM, the results demonstrated significant differences in treatment outcomes across several parameters (Figure 5). For corticosteroid use, BEV monotherapy showed a significant OR of 19.50 (95% CI [2.69, 141.35]; *p* = 0.03), indicating better control over corticosteroid dependency compared to combination therapies. Similarly, for biopsy outcomes, BEV monotherapy had an OR of 18.67 (95% CI [2.55, 136.41]; *p* = 0.04), indicating a more effective reduction in the need for biopsy. For full resection, BEV monotherapy showed a notable OR of 0.05 (95% CI [0.01, 0.39]; *p* = 0.05), suggesting a higher probability of achieving full resection with this treatment. For MGMT methylation status, BEV monotherapy also showed a favorable OR of 43.33 (95% CI [3.90, 481.82]; *p* = 0.05), indicating better epigenetic modulation than combination therapies. Furthermore, the KPS score favored BEV monotherapy, with an OR of 43.33 (95% CI [3.90, 481.82]; *p* = 0.05), reflecting superior performance status. Overall, the total analysis, with a combined OR of 2.24 (95% CI [1.19, 4.20]; *p* = 0.01), showed that BEV monotherapy generally outperformed combination therapies in most parameters. The higher ORs and significant *p* values for BEV monotherapy suggest its superior efficacy in improving clinical outcomes in GBM treatment, particularly by reducing the need for additional interventions such as corticosteroids and biopsies, as well as by improving resection rates and epigenetic responses. The significant heterogeneity across the studies (*I* ^2^ = 77%) emphasizes the need for further research to validate these findings. Additionally, publication bias was assessed using a funnel plot and Egger's test, with a *p* value of 0.02 indicating potential publication bias in the included studies.

### 3.5 TMZ Outperforms CCNU Combo in GBM

**FIGURE 6:**
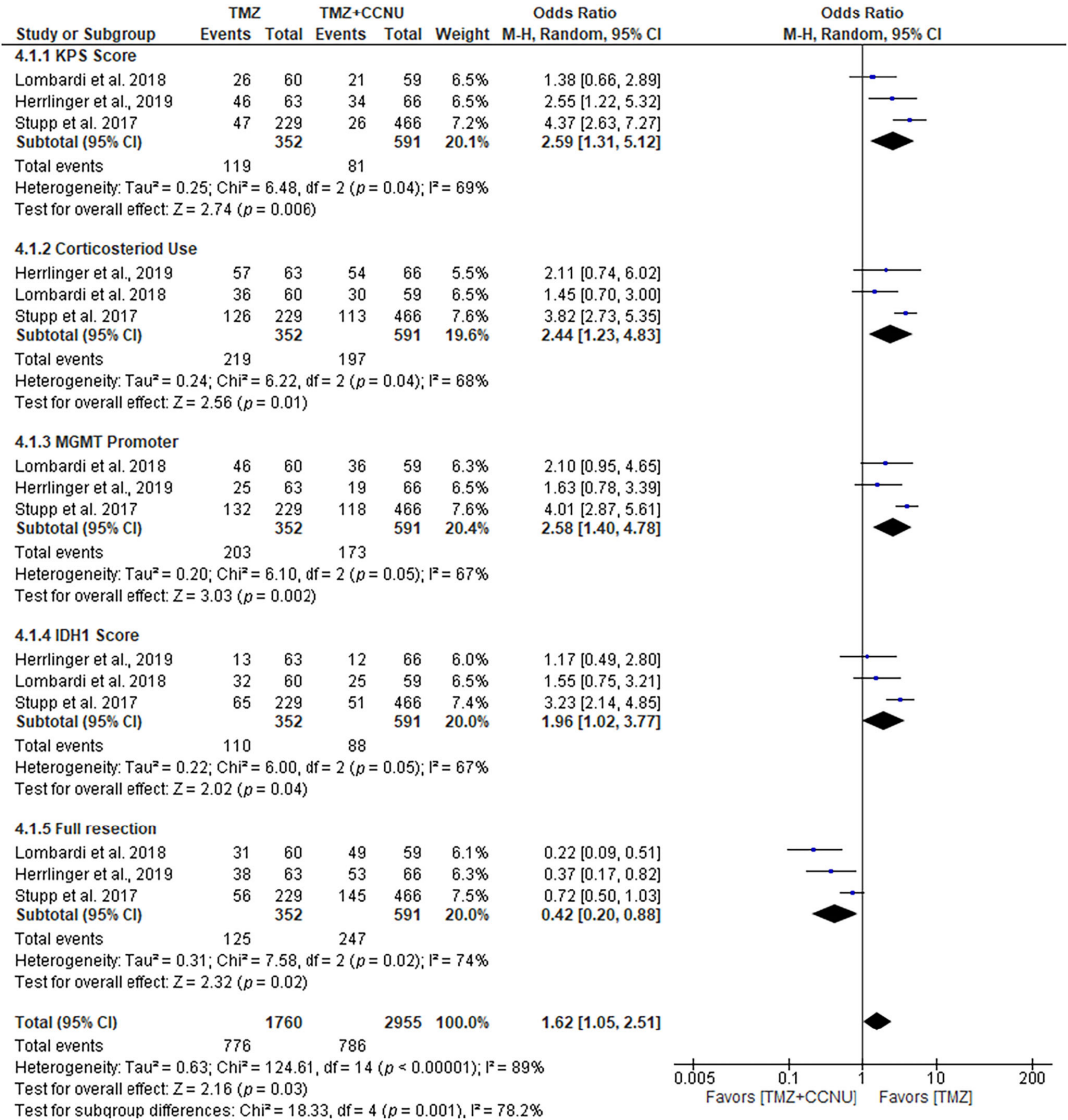
Forest plot of Karnofsky performance (KPS) score, corticosteroid use, and extent of resection among patients treated with temozolomide (TMZ) alone versus combined with lomustine (CCNU). Effect estimates are expressed as odds ratios (OR) with 95% confidence intervals (CI) using a random‐effects model. Statistical heterogeneity was quantified using the *I* ^2^ statistic and Cochran's *Q* test.

In our study comparing CCNU–TMZ (TMZ + CCNU) combination therapy with standard TMZ therapy for the treatment of GBM, TMZ demonstrated superior efficacy across most clinical parameters (Figure 6). The analysis of KPS scores showed TMZ had a significant advantage, with an OR of 2.59 (95% CI: 1.31–5.12, *p* = 0.006), highlighting its greater impact on improving patient performance status compared with combination therapy. Similarly, for corticosteroid use, TMZ was more effective with an OR of 2.44 (95% CI: 1.23–4.83, *p* = 0.01), suggesting a more favorable outcome in reducing steroid dependence. The distribution of MGMT promoter methylation at baseline also differed between arms, with a higher proportion of methylated cases observed in the TMZ arm (OR = 2.58, 95% CI: 1.40–4.78, *p* = 0.002). As MGMT methylation is a pre‐treatment prognostic biomarker rather than a treatment‐induced endpoint, this difference likely reflects baseline cohort characteristics and may partly account for the observed differences in efficacy between arms. In contrast, although TMZ + CCNU demonstrated an advantage in full resection (OR of 0.42, 95% CI: 0.20–0.88, *p* = 0.02), this was the only parameter where the combination therapy outperformed TMZ. Overall, the evidence strongly supports the greater significance and efficacy of TMZ over TMZ + CCNU in the treatment of GBM, as it yielded more consistent and statistically significant improvements in key clinical outcomes, including KPS, corticosteroid use, and MGMT promoter methylation. This suggests that TMZ, as a monotherapy, may be the preferred treatment for most GBM patients, offering a more reliable and effective therapeutic approach. Additionally, publication bias was assessed using a funnel plot and Egger's test. The results from Egger's test (*p* = 0.03) indicated a significant potential for publication bias, suggesting that the observed effects may be influenced by unpublished studies or small sample sizes.

### 3.6 Comparison of Adverse Events Between Standard and Combined Therapies

**FIGURE 7:**
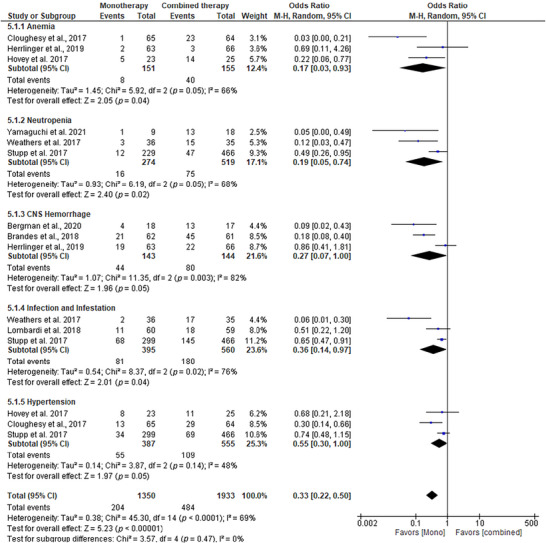
Forest plot of adverse events reported in monotherapy versus combined therapy Effect estimates are expressed as odds ratios (OR) with 95% confidence intervals (CI) using a random‐effects model. Statistical heterogeneity was quantified using the *I* ^2^ statistic and Cochran's *Q* test.

In our study comparing adverse events between standard TMZ monotherapy and combined therapies (TMZ + CCNU) in GBM patients, TMZ monotherapy demonstrated superior efficacy in reducing adverse events across most parameters (Figure 7). In the analysis of anemia, TMZ showed a significantly lower OR of 0.17 (95% CI: 0.03–0.93, *p* = 0.04), suggesting a notable reduction in anemia risk compared to the combination therapy. Similarly, for neutropenia, TMZ exhibited a more favorable profile with an OR of 0.19 (95% CI: 0.05–0.74, *p* = 0.02). For CNS hemorrhage, TMZ demonstrated a better outcome with an OR of 0.27 (95% CI: 0.07–1.00, *p* = 0.05), suggesting that the combination therapy, which includes CCNU, may introduce a higher risk of bleeding or vascular complications. Regarding infection and infestation, TMZ showed a significant advantage, with an OR of 0.36 (95% CI: 0.14–0.97, *p* = 0.04), likely due to the reduced immunosuppression observed with TMZ monotherapy. For hypertension, TMZ also had a better safety profile, with an OR of 0.55 (95% CI: 0.30–1.00, *p* = 0.05), indicating fewer cardiovascular events compared with combination therapy.

The overall pooled OR of 0.33 (95% CI: 0.22–0.50, *p* < 0.00001) underscores the trend that TMZ monotherapy is associated with significantly fewer adverse events. This can be attributed to the fact that the combination therapy, while offering potential benefits in efficacy, may increase toxicity due to the additional chemotherapeutic burden imposed by CCNU. Therefore, TMZ monotherapy not only provides comparable efficacy in treating GBM but also ensures a safer treatment approach with fewer toxicities, making it a preferable choice for managing adverse events in GBM patients. Publication bias was assessed using a funnel plot and Egger's test, which yielded a significant result (*p* = 0.02), suggesting publication bias in the included studies.

#### 3.6.1 PFS and OS Comparison

**FIGURE 8:**
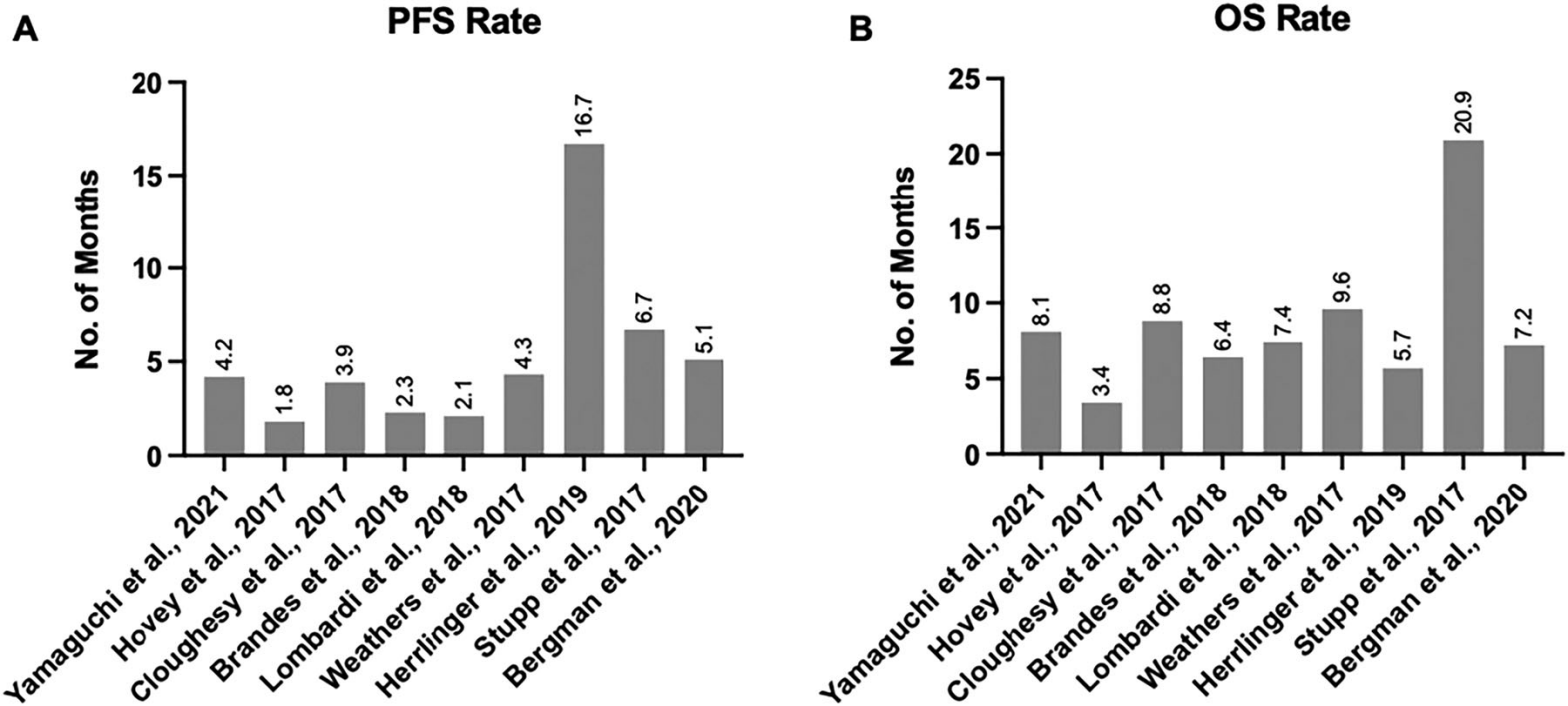
The overall progression‐free survival and overall survival rate reported among included studies. Data are presented as mean values in months. Panel A shows PFS, and Panel B shows OS across included studies. Statistical analysis was performed under a random‐effects model using pooled odds ratios (OR) with 95% CI. OS, overall survival; PFS, progression‐free survival.

**FIGURE 9:**
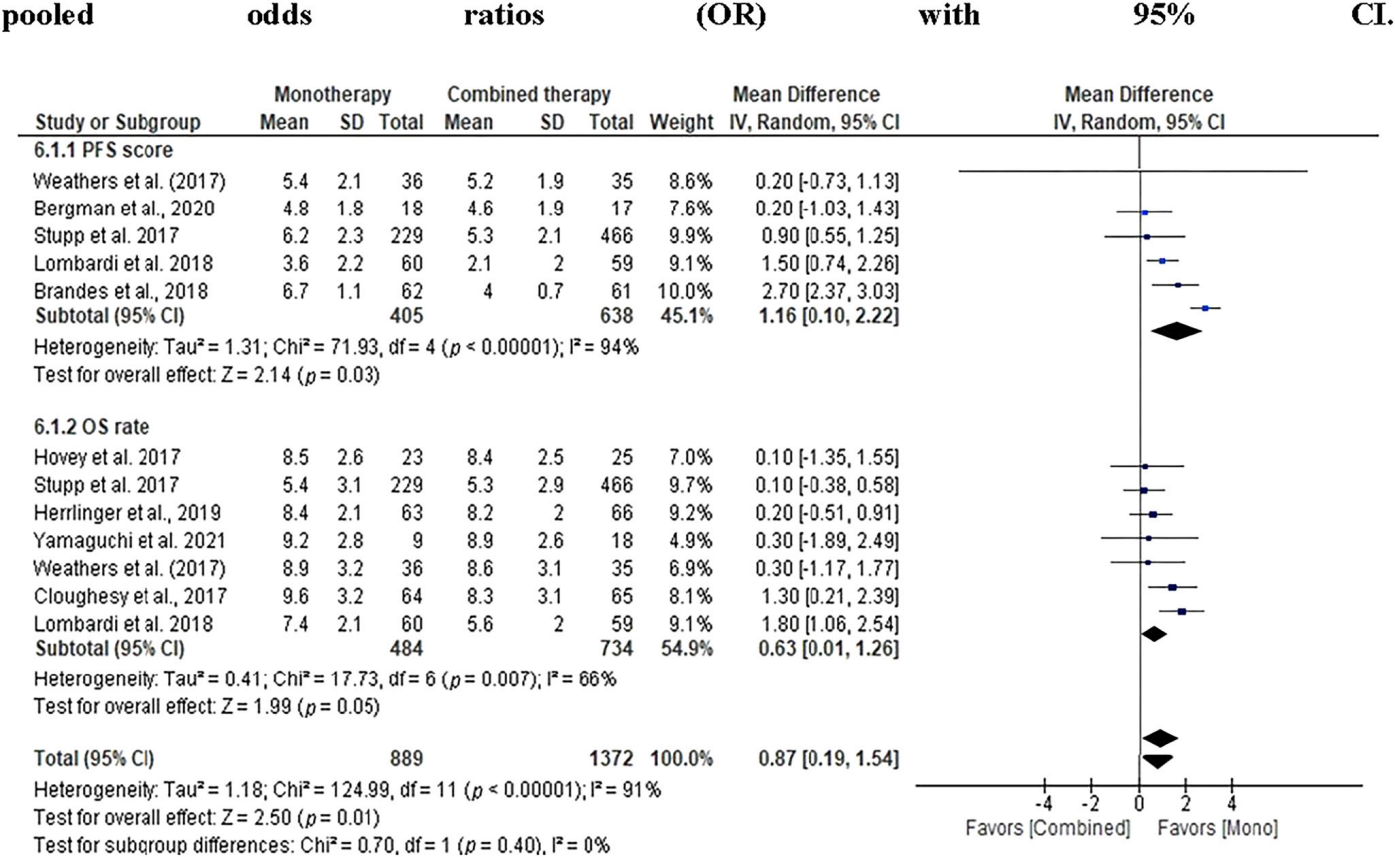
Forest plot of progression‐free survival and overall survival rate among different therapies for treatment of glioblastoma multiforme (GBM). Effect estimates are expressed as odds ratios (OR) with 95% confidence intervals (CI) using a random‐effects model. Statistical heterogeneity was quantified using the *I* ^2^ statistic and Cochran's *Q* test.

In our comparison of PFS and OS between monotherapy and combined therapy, monotherapy showed a significant trend toward superior PFS and OS. The pooled OR for PFS was 1.16 (95% CI: 0.10–2.22), whereas for OS, it was 0.63 (95% CI: 0.01–1.26). Despite the observed heterogeneity across studies, the overall effects for PFS (*Z* = 2.14, *p* = 0.03) and OS (*Z* = 1.99, *p* = 0.05) were marginally significant, suggesting a potential benefit of monotherapy over combined therapy for both survival outcomes. These results suggest that monotherapy may provide better survival outcomes, with a significant trend toward improved PFS and OS, making it a potentially more favorable treatment approach for GBM patients (Figures 8 and 9).

### 3.7 Assessment of Publication Bias and Heterogeneity

The funnel plot was employed to assess publication bias by examining the symmetry of study effect sizes plotted against their SEs. In our analysis, the funnel plot showed slight asymmetry, suggesting potential publication bias among the selected studies. Additionally, moderate heterogeneity was observed, likely due to variations in study designs, sample sizes, and treatment protocols across the studies. This heterogeneity suggests that the observed treatment effects may vary with study‐specific factors, yet the overall findings still provide a meaningful understanding of treatment outcomes for rGBM (Figures S2 and S3).

## 4 Discussion

The study encompassed nine selected articles, scrutinizing various treatment modalities for GBM. Notably, the analysis unveiled nuanced insights into the efficacy of different therapeutic approaches. Comparison of BEV monotherapy versus combination therapies suggested an association with better outcomes for BEV monotherapy, which was associated with more favorable outcomes compared to associations toward better outcomes, particularly in partial resection and Karnofsky performance score (KPS) outcomes (TMZ monotherapy, CCNU combination therapy, corticosteroid use, and KPS). Adverse event analysis revealed higher rates with combined therapies, warranting careful consideration. Moreover, monotherapy showed a trend toward superior PFS and OS compared with combined therapies, albeit not statistically significant, suggesting the potential of monotherapy to optimize GBM treatment strategies (Yamaguchi et al. 2021). These findings underscore the importance of tailored treatment approaches in GBM management and advocate for further investigation to delineate optimal therapeutic regimens.

The clinical heterogeneity across the nine included studies warrants explicit acknowledgment. This heterogeneity is reflected in the high *I* ^2^ values observed throughout (up to 86%) and means that pooled effect estimates must be interpreted with caution. Subgroup analyses were performed where possible, and sensitivity analyses excluding high‐risk‐of‐bias studies were conducted; however, the small number of eligible studies limits the statistical power of these explorations. The observed associations should therefore be viewed as preliminary signals requiring prospective validation rather than definitive evidence of treatment superiority. Managing rGBM presents challenges due to the lack of standard treatment. Though cytoreductive surgery improves survival, it is underused. Predictors of post‐surgery survival include age, performance status, interval between surgeries, tumor size, and resection extent (Hovey et al. 2017). However, our study identified a postoperative decrease in KPS as a significant clinical factor affecting OS after recurrence, highlighting the complexity of prognostic factors in this context (Cloughesy et al. 2019). Prior to the approval of BEV, the prognosis for rGBM patients treated with the Stupp protocol was modest, indicating the necessity for additional treatment modalities (Stupp et al. 2017). Following BEV approval, there was a notable improvement in patient prognosis, particularly when BEV was combined with cytoreductive surgery and palliative chemotherapy. This observation aligns with previous retrospective studies demonstrating prolonged survival with this combination (Brandes et al. 2019). Furthermore, our study underscores the potential of BEV therapy to improve outcomes in rGBM, consistent with similar investigations reporting survival benefits with BEV‐containing regimens. Importantly, BEV monotherapy exhibited promising efficacy compared to combination therapies in GBM treatment, highlighting its significance as a valuable therapeutic option (Bergman et al. 2020). Additionally, exploring biomarkers such as HGF expression and MGMT methylation status may provide valuable insights into patient stratification and the prediction of treatment response, although further prospective studies are necessary to validate these findings (Gilbert et al. 2014). In summary, our study advances understanding of treatment strategies for rGBM and underscores the potential of BEV‐based therapies to enhance patient outcomes (Lombardi et al. 2019).

Our study echoes prior research on BEV in recurrent glioblastoma (GBM), showing improved (PFS) but no clear (OS) advantage. Despite BEV's potential, its impact on OS remains uncertain, consistent with combined chemotherapy trials (Weathers et al. 2016). Interestingly, BEV‐treated patients in our study had a shorter median OS than BEV‐naive patients, possibly due to prior BEV exposure. Investigating BEV with other agents, such as CCNU, yielded mixed results, consistent with our modest OS benefits observed with BEV alone (Lombardi et al. 2017). However, our study also highlights limited OS improvements with TMZ, despite its efficacy in reducing corticosteroid use, consistent with AVAglio findings (Herrlinger et al. 2019).

Moreover, our study emphasizes the nuanced evaluation of adverse events associated with these therapies. Although BEV showed a manageable safety profile consistent with previous studies, combinations, such as CCNU plus BEV, were associated with higher rates of severe adverse events, including thrombocytopenia (Chinot et al. 2014). Similarly, regorafenib demonstrated notable improvements in PFS and OS but was associated with higher rates of Grade 3–4 adverse events compared with CCNU (Lombardi et al. 2019). These findings underscore the complexity of treating rGBM and highlight the need for further research to optimize therapeutic strategies, balancing efficacy with adverse event management to enhance patient outcomes (Taal et al. 2014).

Discussing the cost‐effectiveness of treatments for GBM is crucial for making informed healthcare decisions. The use of economic analysis to guide patient care is most consistent with the ethical principle of justice in society but may conflict with the other ethical principle of beneficence for the individual patient with complex, rare, or serious diseases (Balak et al. 2020). At this juncture, the physician can significantly contribute to enhancing the efficiency, fairness, and quality of healthcare while maintaining the provision of effective services. Essentially, more efficient healthcare means better care for individual patients and requires increasing the available resources to improve care for the entire population. Hidden value judgments that allow rationing under the camouflage of prioritization, hidden conflicts of interest between societal and individual patient needs, and biases in the application of medical tools can compromise the responsible and optimal allocation of resources (Eijkholt et al. 2021). Therefore, it is necessary to be aware of these pitfalls to distribute patient care transparently and accountably.

Medical and surgical innovations in the treatment of rGBM continue to evolve because there is currently no consensus on how best to manage relapse and uncertainty. Patients with rGBM have the right to benefit from innovative surgical treatments in line with the fundamental ethical principle of patient autonomy (Cote et al. 2017). However, reasonable and appropriate precautions must be taken to ensure adequate protection of these vulnerable patients. First, a high standard of informed consent must be obtained, with particular attention to the patient's capacity to consent. Second, significant oversight and regulation of innovative treatments are required. Third, there must be sufficient evidence in published human or animal model studies that the relevant innovative treatment works. Fourth, there should be no risk of harm to the patient. If these standards are not met, the patient's right to innovative treatment is violated. TMZ combined with RT has been shown to improve survival outcomes but comes with significant costs. Studies indicate that although TMZ is effective, its high cost must be weighed against its clinical benefits (Hovey et al. 2017) (Lombardi et al. 2017). BEV has been used for rGBM, and although it shows promise in improving PFS, its OS benefits remain unclear. BEV treatment is also associated with high costs and increased adverse events, necessitating a careful cost‐benefit analysis (Herrlinger et al. 2019; Chinot et al. 2014). Cost‐effectiveness analyses emphasize the need for personalized treatment plans that optimize both economic and clinical outcomes. Future research should continue to explore the economic implications of these therapies to provide comprehensive guidelines for GBM treatment (Farooq et al. 2023; Encarnacion‐Santos et al. 2024; Ahmed et al. 2024; de Sena Barbosa et al. 2024).

The strength of our meta‐analysis lies in its comprehensive synthesis of existing literature, elucidating the efficacy and safety profiles of various treatment modalities for rGBM, including BEV and TMZ, both as monotherapies and in combination regimens. By examining contrasting findings from multiple studies, we provide valuable insights into the nuanced outcomes associated with different treatment approaches, highlighting the need for personalized therapeutic strategies in managing rGBM. However, the limitations of our meta‐analysis include reliance on retrospective studies with varying sample sizes and methodologies, which necessitates caution in interpreting the pooled results and underscores the need for further prospective research to validate our findings and guide clinical decision‐making effectively. Importantly, the Cochrane risk of bias assessment revealed that the included studies carry high risk of bias in multiple domains—particularly incomplete outcome data, selective reporting, and other biases—with only Weathers et al. (2020) and Stupp et al. (2017) rated as low risk across most domains (Table 1). This pervasive high risk of bias substantially limits the certainty of the pooled estimates and means that results should be regarded as hypothesis‐generating rather than definitive. Furthermore, Egger's test yielded statistically significant asymmetry for several analyses (*p* = 0.02–0.03), indicating potential publication bias that may have inflated effect size estimates. These methodological concerns are reflected in the wide CI and high heterogeneity (*I* ^2^ up to 86%) observed throughout and together warrant considerable caution.

## 5 Conclusion

Our meta‐analysis provides valuable insights into the efficacy and safety of various treatment options for rGBM. Among the therapies reviewed, BEV monotherapy stands out as a promising option, particularly in terms of KPS results, though its OS benefits remain inconclusive. Further research Specifically, while BEV monotherapy was associated with marginally significant OS benefit compared to pre‐BEV regimens in one analysis (OR = 0.63, *p* = 0.05), this finding was not replicated across all comparisons, and the OS advantage of BEV did not reach conventional significance thresholds when accounting for heterogeneity. Similarly, the superiority of TMZ over TMZ + CCNU observed in several parameters must be interpreted in the context of Herrlinger et al. (2019), which specifically demonstrated an OS benefit for the CCNU–TMZ combination in newly diagnosed, MGMT‐methylated GBM, a distinct population from rGBM. These results are therefore not contradictory but reflect the different patient populations studied which is necessary to delineate the specific advantages of BEV and optimize its therapeutic application. Future studies should address gaps in the current literature, such as the long‐term effects of BEV and other monotherapies, by employing large‐scale, prospective, RCTs. Additionally, exploring biomarkers for patient stratification and response prediction could enhance personalized treatment strategies. Investigating the cost‐effectiveness of these therapies is also crucial for providing a comprehensive framework for clinical decision‐making. By focusing on these areas, future research can advance the field and improve outcomes for GBM patients.

## Author Contributions

**Kayode Agboola**: conceptualization, writing – original draft, writing – original draft, investigation, formal analysis. **Fatemeh Khafaji**: software, formal analysis. **Bipin Chaurasia**: writing – review and editing, visualization, validation, supervision. **Kashif Qureshi**: writing – review and editing. **Kivanc Yangi**: writing – review and editing. **Vishal Chavda**: methodology, validation, data curation. **Naci Balak**: visualization, investigation, supervision.

## Conflicts of Interest

The authors declare no conflicts of interest.

## Funding

The authors have nothing to report.

## Ethics Statement

The authors have nothing to report.

## Supporting information




**Supplementary Figures**: brb371456‐sup‐0001‐FigureS1‐S3.docx

## Data Availability

All data are publically available within manuscript.
