# Supplementary material for: Advancements in Glioblastoma Multiforme Treatment: A Comprehensive Systematic Review and Meta‐Analysis
Source: Brain Behav. 2026 Apr 28;16(5):e71456. doi: 10.1002/brb3.71456 (PMC13125373; doi:10.1002/brb3.71456)
Supplement: Supplementary file 1 — Supplementary Figures: brb371456‐sup‐0001‐FigureS1‐S3.docx [file BRB3-16-e71456-s001.docx]

**Advancements in Glioblastoma Multiforme Treatment: A Comprehensive Systematic Review and Meta-Analysis**

**Supplementary Figures**


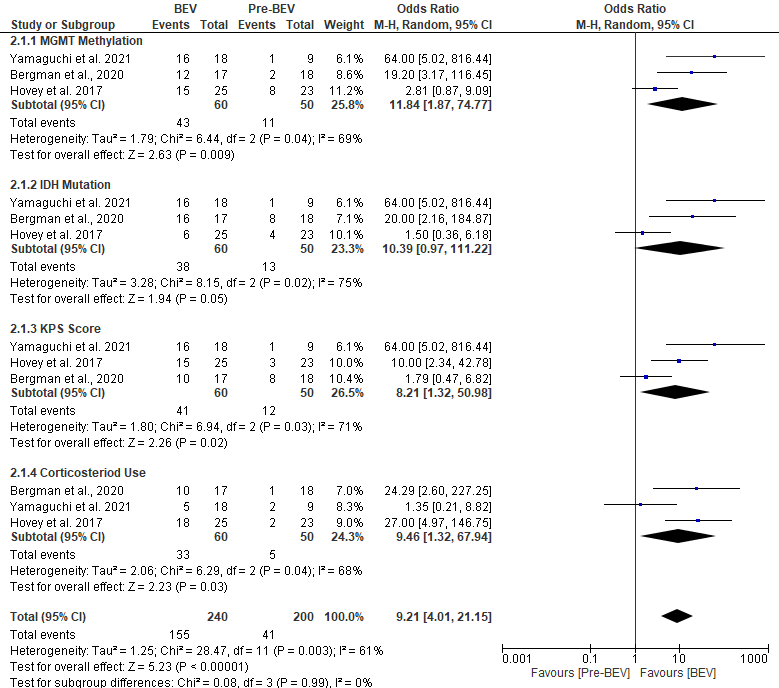


**Supplementary Figure 1.** Forest plot of baseline biomarker distribution (MGMT promoter methylation, IDH mutation status, Karnofsky Performance Score, and corticosteroid use) across Pre-Bevacizumab and Bevacizumab-treated patient groups. These variables represent pre-treatment prognostic characteristics and were compared using odds ratios (OR) with 95% confidence intervals (CI) under a random-effects model. Statistical heterogeneity was assessed using the I² statistic and Cochran’s Q test. These biomarkers are established pre-treatment prognostic indicators and do not represent treatment-induced endpoints. Differences likely reflect baseline cohort imbalances rather than biological effects of therapy.


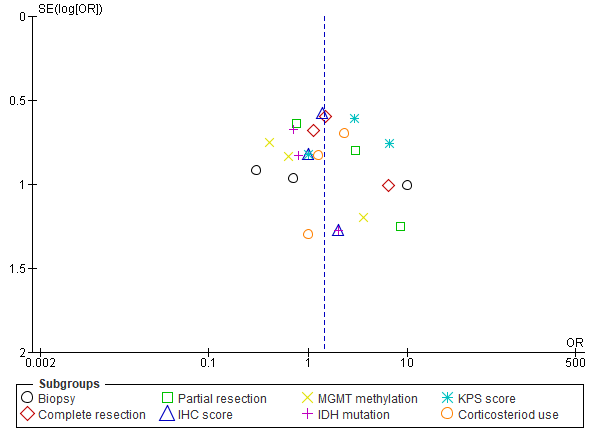


**Supplementary Figure 2.** Funnel plot of effect size versus standard error for studies comparing Pre-Bevacizumab versus Bevacizumab-treated patients, used to assess publication bias. In the absence of publication bias, study results are expected to distribute symmetrically about the pooled effect size forming an inverted funnel. Asymmetry was evaluated using Egger’s regression test (P = 0.15), indicating no significant publication bias for this comparison.


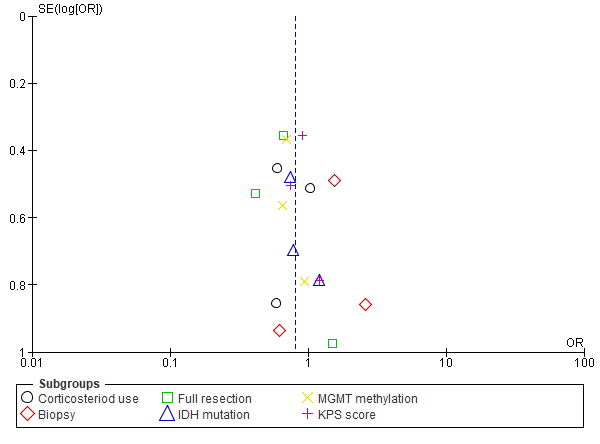


**Supplementary Figure 3.** Funnel plot of effect size versus standard error for studies comparing Bevacizumab monotherapy versus combined therapy in recurrent GBM patients, used to assess publication bias. Asymmetry was evaluated using Egger’s regression test (P = 0.02), indicating potential publication bias that may have inflated the observed effect size estimates for this comparison. Results from this analysis should be interpreted with appropriate caution.
